# The effect of blind box product uncertainty on consumers’ purchase intention: The mediating role of perceived value and the moderating role of purchase intention

**DOI:** 10.3389/fpsyg.2022.946527

**Published:** 2022-08-03

**Authors:** Yi Zhang, Tianqi Zhang

**Affiliations:** School of Economics and Management, Shanghai Institute of Technology, Shanghai, China

**Keywords:** blind box, uncertainty, perceived value, purchase intention, customers purpose

## Abstract

As the younger generation, who like to pursue novelty and excitement, becomes the main consumer and the traditional consumption culture changes in China, the blind box has become a popular product among young people with its uncertain characteristics. Previous studies have mainly explored the role of uncertainty in promotion, while this paper focuses on the role of uncertainty in daily sales of blind box products. Based on the stimulus–organism–response (SOR) theory, this paper conducted an online questionnaire survey and an empirical analysis in China, which examined the mechanism of the positive impact of uncertainty and the moderating effect of consumption purpose. The results show that uncertainty affects consumers’ purchase intention mainly through affecting their emotional value, which is one dimension of perceived value; consumer purpose also moderates the effect of uncertainty on perceived value, and the effect of perceived value on purchase intention. The results of this study are not only of great significance for understanding the uncertain marketing and blind box products, but also have management implications for enterprises to make use of the uncertain marketing.

## Introduction

According to Ali Research, China’s older generation (over 35 years) spent $1.9 trillion, while the new generation (18–35 years) spent $1.5 trillion in 2016. China’s older generation (over 35 years) consumed US $2.4 trillion, while the new generation (18–35 years) consumed US $2.6 trillion in 2020. From 2016 to 2020, the consumption of young consumers increased by 73%. Meanwhile, the consumption of young consumers has surpassed the consumption of those over 35 years old to become the main consumption force by 2020. Compared with their parents, post-90s consumers have greatly improved material conditions, which makes them have more personalized consumer demands, preferring novelty and stimulation (Wang and Zhou, 2021). Therefore, blind boxes have become popular products among young people with their uncertain characteristics.

The concept of the blind box originated in Japan. It refers to a closed, opaque small box with the same exterior packaging and built-in dolls of different styles. In China, blind boxes mainly include dolls, such as Molly and Pucky, as well as animation and film co-branded blind boxes, such as Toy Story and Disney. In recent years, due to the blind box enterprises represented by POP MART regularly launching exquisite and diverse products, offline automatic box pumping machines are all over major shopping malls, which makes blind boxes popular among young consumers with their features of exquisite design and high uncertainty. There is solid evidence that the blind box has become the most popular product in the fashion consumption of the Chinese young generation. According to the “List of post-95 players hands-chopping” released by Tmall, among the most “expensive” hobbies of “post-95,” garage kit ranked the top, among which blind box became the field with the fastest-growing number of players. According to research data, the market size of the blind box industry in China is expected to reach 25 billion yuan in 2025 (Zeng, 2021). The popularity of blind boxes lies in the word “blind,” that is, uncertainty (Yan and Wu, 2021).

However, traditional research generally believes that consumers tend to pursue certainty rather than uncertainty. In addition, traditional Chinese culture advocates objecting to extravagance and praising austerity, so consumers will make more cautious choices in consumption and tend to avoid risks (Zhou, 2013). Therefore, many academic studies have focused on reducing or eliminating consumers’ perceptions of uncertainty. For example, Taobao sellers reduce consumers’ perceived uncertainty through online communication and buyer evaluation (Zhang and Liu, 2010), which can reduce the commodity return rate by reducing uncertainty (Matt and Hess, 2016). Brand reduces perceived uncertainty through brand community, so as to increase customer fit and brand loyalty (Fan and Chai, 2017).

Recently, the positive effects of uncertainty have received increasing attention. Goldsmith and Amir (2010) believe that taking uncertainty into account in marketing is a method to reduce marketing costs and maintain consumers’ interest, which has a good marketing effect. Shen et al. (2015) argued that people would invest more time, money, and energy to obtain uncertain rewards. Based on the positive emotion theory, Lee and Qiu (2009) proposed that when consumers face an uncertainty related to a positive event, they will experience greater and more lasting positive emotions. Shou and Zheng (2017) sorted out nearly 20 years of research on uncertain promotion and paid attention to the positive role of uncertain betting games in service recovery (Shou et al., 2022). In practice, an increasing number of enterprises begin to make use of uncertain marketing, For example, online merchants provide uncertain freebies to increase customers’ happiness (Zhang et al., 2021); the emergence and popularity of blind box and its derivative products (Yan and Wu, 2021).

Although some scholars have discussed the positive effect of uncertainty on consumers’ purchases, most of the above literature focuses on the design of uncertain gifts or promotional activities, while the blind box, in which the uncertainty factor itself is the selling point, does not involve gifts and promotional activities. Due to the explosion of blind boxes in China, the existing relevant studies are all in China, while most of the existing studies on the blind box by Chinese scholars are from a qualitative perspective, studying the consumption mentality, emotional needs, and cultural behaviors of blind box players (Wang and Zhou, 2021; Zeng, 2021). Quantitative studies have explored the impact of different components of customer experience on blind box repeat purchases (Yan and Wu, 2021). There is no clear answer on how uncertainty affects consumer purchase. As a new fashion toy, why does the uncertainty of blind boxes appeal to consumers? What kind of experience does uncertainty bring to consumers? How do these experiences affect consumer decision-making? There are no clear answers to these questions.

Based on the existing research, this paper uses a questionnaire survey to conduct empirical research that explores the influencing factors of uncertainty driving consumers to buy blind boxes, focusing on the mediating role of perceived value between uncertainty and blind box purchase intention. The results contribute to a better understanding of the positive effects of uncertainty and blind box products.

## Literature review

### The positive effects of uncertainty

Some scholars have studied the mechanism of uncertainty marketing, in which summarized theoretical basis mainly includes positive emotion, information gap theory, uncertainty resolution, and innate optimism theory.

First, uncertainty priming induces a higher arousal state, which increases physiological arousal, which in turn makes people experience their emotions more intensely (Ruan et al., 2018). Positive uncertain events have been found to be imagination-provoking to the point that it is more likely to obtain and maintain positive emotions (Lee and Qiu, 2009). Meanwhile, Ketelaar et al. (2018) believes that positive uncertainty increases consumers’ positive feelings when evaluating products, especially for high-level products, so that consumers can imagine and speculate on potential enthusiasm. When uncertainty is related to positive events, consumers may prefer to choose uncertain products and services (Lee and Qiu, 2009). For example, people will have higher expectations when the movie trailer they watched is uncertain about the specific content and the ending compared to when it is certain (Wilson et al., 2005).

Second, the information gap theory holds that there will be an information gap between the known information of a person and the unknown and desired information, which leads to a strong desire for information (Loewenstein, 1994). For example, ambiguous advertisements will make consumers desire complete information, arousing curiosity, and attracting more attention (Daume and Hüttl-Maack, 2019).

Furthermore, according to the information gap theory, Hsee and Ruan (2016) proposed the uncertainty resolution theory, which holds the view that human beings are born with an inherent desire to solve uncertainty. Even if the content expectation is negative, people may feel temporarily relaxed when solving uncertainty. For example, in advertising marketing, compared with receiving all information at the same time, consumers who receive partial missing information and then obtain complete information will have a better enjoyment experience (Ketelaar et al., 2018). In research on the mechanism of uncertainty reinforcement repetition, Shen et al. (2019) believed that uncertainty resolution is a psychological reward, which can be regarded as positive reinforcement.

Furthermore, the innate optimism theory suggests that many people subconsciously perceive themselves as lucky, estimating outcomes optimistically. Based on this theory, Ailawadi et al. (2014) concluded that in the face of uncertain promotion, people will think that the probability of favorable results is greater, so uncertain marketing has a better effect; Goldsmith and Amir (2010) found that consumers choose free mystery rewards (as opposed to known rewards) because they are too optimistic about the nature of free gifts.

Finally, customers’ purchase purpose and product type also influence the effect of uncertain marketing. Laran and Tsiros (2013) believed that when an emotional purchase is involved, more attention has been paid to experience and emotional factors, and thus, it is more likely that customers express greater interest and motivation toward uncertain marketing. When it comes to cognitive purchase, more clear and detailed information should be provided to make decisions, and thus, it is more likely that customers prefer deterministic factors. At the same time, Goldsmith and Amir (2010) proves that different categories of commodities will affect consumer focus, which leads to the difference in attitude and behavior. When the target product is practical, consumers will consider the product itself more and pay more attention to the certainty information when making decisions; while when the product is hedonic, consumers will consider more emotional factors when making decisions, and uncertainty is more likely to play a positive role.

Chinese scholars Shou and Zheng (2017) summarized the information gap theory, positive emotion theory, and innate optimism theory when sorting out uncertain promotion strategies and also recognized the influence of customers’ purchase purpose and product type. Positive emotion theory is the most widely used in uncertainty application research. Yan and Wu (2021), when studying the effect of blind box purchase customer experience on customer happiness, verified that unpredictability would produce positive emotions such as curiosity and adventure, thus bringing customer happiness according to the positive emotion theory. Shou et al. (2022) believe that uncertain betting games can bring positive emotional experiences, such as surprise, feeling of winning, and a sense of fun, and can hedge customers’ negative emotional experiences in service failure to a certain extent and improve customer satisfaction, so as to realize service recovery. Jin and Wang (2019) summarized the theoretical basis of rewarding uncertain utility through information gap theory, positive emotion theory, and optimism theory when analyzing the influence of game elements on consumer engagement behavior.

### Perceived value

Perceived value refers to “consumers’ overall evaluation of product utility, which is based on consumers’ perception of benefits and sacrifices of products” (Sheth et al., 1991). A large number of consumer studies have shown that perceived value is the key factor that directly affects customers’ purchase intention. Customer perceived value can be divided into many dimensions, and different scholars put forward different dividing ideas according to their research background. Sheth et al. divided perceived value into five levels, including functional value, social value, emotional value, conditional value, and epistemic value, and believed that functional value was the decisive factor in consumer decision-making (Sheth et al., 1991). When studying the factors influencing online consumers’ purchasing decisions, Li et al. (2017) divided perceived value into product perceived value, social perceived value, and service perceived value. By analyzing the determinants of perceived value, Liu (2008) divided it into functional value, emotional value, and social value. Based on the interview data and online comments, this paper summarizes the features of blind boxes, such as the novelty of appearance, excitement, and sociability, and refers to the last classification basis.

### Stimulus–organism–response theory

Mehrabian and Russell (1974) proposed the SOR (stimulus–organism–response) theory, which holds that the environment, as a stimulus, will cause emotional changes and ultimately affect individual behaviors. SOR theory has been widely used in the research of consumer behavior. Among them, “stimulus” refers to various environmental factors affecting customer cognition; “organism” refers to customer perception (cognition, emotion, etc.); “response” includes customer purchase, recommendation, adoption, and other behaviors (Uzzi, 1996). For example, in the online shopping environment, uncertain factors (S) such as online shopping platforms and commodity information will affect consumers’ internal perception (O) of online shops and commodities, and ultimately, affect their decision-making (R) (Sevgin et al., 2003). From the perspective of reducing uncertainty, Kaur et al. (2017) regarded brand familiarity and supplier brand reputation as stimuli (S), which could influence trust and attitude (O) and ultimately purchase intention. According to the information gap theory, uncertainty, as an external environment, can arouse individual behavior (Loewenstein, 1994). Negative uncertainty may cause psychological discomfort, anxiety, and other emotions, while positive uncertainty may generate more challenges and excitement, as well as create imagination space for customers and lead to more positive emotions (Lee and Qiu, 2009). Therefore, this paper considers uncertainty as a stimulus in the blind box purchase scenario, which will further affect consumers’ internal perception.

Zeithaml (1988) believed that perceived value is a subjective judgment made by users in the process of purchasing products or enjoying services. Therefore, many researchers regarded perceived value as an organism. For example, Li Chuang et al. (2021) believed that consumption promotion policies (S) of new energy vehicles would affect the perceived value and perceived risk (O), and ultimately affect purchase intention (R); Chopdar and Balakrishnan (2020) used perceived value and impulsivity as an organism in exploring the drivers of buyback intention and satisfaction experience in the mobile commerce shopping environment (O). In the blind box purchase scenario, perceived value can also appropriately reflect the impact of uncertainty on consumers, which made us choose it as an organism (O). Consumers with high perceived value will make further purchases. Based on the above analysis, this paper selects the SOR theory as the theoretical framework, the uncertainty as an antecedent variable, perceived value as an intermediary variable, purchase intention as the outcome variable, and purpose of consumption as the moderating variable between uncertainty and perceived value; and perceived value and purchase intention, to explore the internal mechanism of uncertainty affecting blind box purchase intention.

## Research hypotheses and conceptual models

### Uncertainty, functional value, and purchase intention

The information abundance of products will have an important impact on the experience and further affect customers’ judgment of perceived value (Chen and Dubinsky, 2003). Duan et al. (2012) also hold that perceived risk has a negative impact on perceived value. In the process of blind box purchase, due to the uncertainty caused by the opaque packaging, the product visual information and quality information cannot be directly displayed in front of consumers, which makes it difficult for consumers to judge whether the style of personalized products is suitable for themselves and doubt the reliability of products; therefore, it will have a negative impact on the functional value of the product.

Functional value refers to the perceived utility of a product from practicality, utility, or physical ability (Sheth et al., 1991). Traditional research believes that this is the original intention of consumers to make purchase decisions. In this study, the beautiful and personalized product design of the blind box reflects the functional value. Product visual information can establish the first impression of products to consumers, and with the homogenization of product attributes and the intensification of market competition, product appearance novelty has become an important means of differentiation (Talke et al., 2017). The visual stimulation of “beauty” will activate the reward center in the brain, thus affecting people’s judgment and decision-making (Mugge et al., 2017). Therefore, the aesthetic experience obtained by consumers from vision will affect their behavior, such as showing off aesthetic products, cherishing products more, and being more willing to buy (Yan and Wu, 2021). A blind box is usually a cartoon image or cartoon character designed by a well-known designer. Its exquisite design will make consumers desire to buy it the moment they see the picture on an online platform or the outer package of the blind box. Based on the above discussion, the following hypothesis is proposed:

H1a: Uncertainty has a negative effect on functional value.
H2a: Functional value positively affects purchase intention.

### Uncertainty, emotional value, and purchase intention

Emotional value refers to the satisfaction consumers get in emotional experience while enjoying products and services (Sheth et al., 1991). The uncertainty of the blind box will promote consumers to generate positive emotions and emotional value.

More and more studies have found that uncertainty is a state of arousal, which can arouse consumers’ curiosity (Loewenstein, 1994) and imagination space (Lee and Qiu, 2009). At the same time, it brings positive experiences such as surprise (Laran and Tsiros, 2013), excitement, and stimulation (Lee and Qiu, 2009). On the one hand, curiosity is a state of information deprivation. When deprivation is solved, people will experience a happy sense of relief (Loewenstein, 1994); on the other hand, curiosity is a sense of interest, which is more consistent with the concept of positive emotion (Ruan et al., 2018). In addition, curious consumers understand that uncertainty will be solved soon, resulting in positive expectations. In fact, people can get a pleasant experience of uncertainty resolution at the moment of uncertainty resolution (Shen et al., 2019). The opaque packaging of the blind box will arouse the curiosity of players. In addition, the blind box merchants usually display the specific design of each product on the online platform. When consumers see the beautifully designed pictures, they will have imagination space and further generate positive emotions, such as expectation and excitement.

Positive emotions are extremely important to customer satisfaction and customer purchase (Laran and Tsiros, 2013). Consumers mainly make shopping decisions through rational thinking and emotional experience, that is, the descriptive attribute information of the product and the subjective emotional feelings related to the product are the basis for consumers to make purchase decisions (Iyer et al., 2019), while the uncertain priming increases the impact of emotional input on consumers’ judgment and decision-making, making consumers pay more attention to the emotional attributes of the product (Faraji-Rad and Pham, 2017). In addition, the mere state of uncertainty can stimulate hedonic consumption to some extent (Rauwolf et al., 2021). Based on the above discussion, the following hypotheses are proposed:

H1b: Uncertainty has a positive effect on emotional value.
H2b: Emotional value positively influences purchase intention.

### Uncertainty, social value, and purchase intention

Social value focuses on improving consumers’ sense of gain at the social level (Sheth et al., 1991). Blind box uncertain gameplay can bring to the player social value (Yan and Wu, 2021).

According to the uncertainty resolution theory, players can solve the uncertainty after opening the box as a psychological reward (Shen et al., 2019). Different types of rewards can strengthen promotion and innovation, thus generating and creating a kind of behavior repetition (Koo and Fishbach, 2010). Different purchasing experiences of players will lead to differences in sharing intentions. The reward of uncertainty resolution will increase the positive experience of sharers and further promote sharing. Furthermore, due to the stimulation and interest in uncertain rewards, customers’ willingness to recommend uncertain rewards is higher than that under specific rewards (Wang et al., 2018). In addition, the common interests, preferences, and pursuit of certain values of consumers are the bridges of social interaction. Players connect originally isolated individuals through the common love of uncertainty pumping. In the process of players’ close interaction, a large number of highly valuable product information will also be generated, and the dissemination and sharing of such information will affect consumers’ shopping behaviors (Wang and Ping, 2012). Based on the above discussion, the following hypotheses are proposed:

H1c: Uncertainty has a positive impact on social value.
H2c: Social value positively influences purchase intention.

### The moderating effect of consumption purpose

In this paper, consumption objectives are divided into pursuit process and pursuit result (Shen et al., 2015). When people pursue a process, positive experience is crucial, and thus, it is possible that completion and emotional enrichment increase motivation (Fishbach and Choi, 2012). In contrast, when people pay more attention to the results and have a corresponding expectation of returns, they will be more cautious, and thus, it is possible that profitability and negative impact are the core of action evaluation (Hsee and Rottenstreich, 2004). Specifically in a blind box purchase, that is, process-oriented blind box buyers enjoy the experience of coming out and unpacking blind boxes, enjoying the challenge, excitement, and excitement of uncertainty. Therefore, uncertainty has a greater positive impact on emotional value as well as emotional value has a greater impact on purchase intention. At the same time, blind box buyers who pursue results may have a strong purpose for a certain doll because they like the appearance and design of the doll or want to collect all the dolls. Therefore, uncertainty has a greater negative impact on the functional value as well as the functional value has a greater impact on the purchase intention.

Considering that blind box consumers have the above two different consumption purposes, the following hypotheses are proposed:

H3a: Consumption purpose regulates the relationship between uncertainty and functional value, that is, compared with consumers pursuing process, uncertainty has a greater negative effect on functional value.
H3b: Consumption purpose regulates the relationship between uncertainty and emotional value, that is, compared with consumers pursuing results and consumers pursuing process, uncertainty has a less positive effect on emotional value.
H3c: Consumption purpose regulates the relationship between uncertainty and social value, that is, compared with consumers pursuing results and consumers pursuing process, uncertainty has a less positive effect on social value.

H4a: Consumption purpose regulates the relationship between functional value and purchase intention, that is, functional value has a greater positive effect on purchase intention than consumers pursuing results and process.
H4b: Consumption purpose regulates the relationship between emotional value and purchase intention, that is, compared with consumers pursuing results and consumers pursuing process, emotional value has a less positive effect on purchase intention.
H4c: Consumption purpose regulates the relationship between social value and purchase intention, that is, compared with consumers pursuing results and consumers pursuing process, social value has a less positive effect on purchase intention.

**FIGURE 1:**
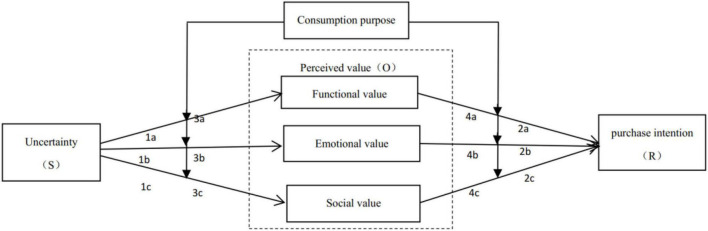
Hypothetical model.

See Figure 1 for the specific model.

## Research design

### Variable measurement

In this study, the antecedent variable is uncertainty; the mediating variable is the three dimensions of perceived value: functional value, emotional value, and social value; the dependent variable is purchase intention; and the moderating variable is consumption purpose. Among them, uncertainty is adapted from the measurement scale proposed by Zhang and Liu (2010), including three items. Perceived value refers to the scale of Sweeney and Soutar (2001) and is adapted according to the comments of players in the blind box community, POP MART WeChat official account, and other channels. Functional value, emotional value, and social value all have three or more items. Purchase intention refers to the viewpoint of Dodds et al. (1991), with a total of four items. Consumption purpose is divided into process-oriented and results-oriented (Shen et al., 2015).

### Research process and data collection

This study designed the questionnaire through the questionnaire star. Since the study focused on the role of uncertainty in blind box consumption, we required all the participants to have experience in blind box purchase. The questionnaire was released in the blind box exchange WeChat group, the blind box player QQ group, Xianyu blind box community, the Douban POP MART group, and other domestic blind box communities, which have a great many blind box players. To further confirm that every respondent has blind box consumption experience, the first question of the questionnaire is designed as “do you have blind box purchase experience,” and only those who fill in “yes” can continue to answer the following questions. A total of 407 questionnaires were collected, and a total of 375 valid questionnaires were obtained, with effective recovery of 92.1%, excluding those with too short response time and obvious response regularity. In terms of sample characteristics, there are more female samples; most of the age groups are 18–25 years old. The education level is mainly undergraduate and junior college students; the monthly income of more than 5,000 yuan accounted for 46.9%. Specific sample characteristics are shown in Table 1.

**TABLE 1:** Sample overview (*N* = 375).

Variable	Category	Percentage (%)	Variable	Category	Percentage (%)
Gender	Male	35.7	Education degree	High school and below	2.9
	Female	64.0		Junior college	25.1
Age	<18	1.6		Undergraduate	65.9
	18–25	58.4		Master degree or above	6.1
	26–30	34.7	Monthly income	<2,000¥	12.3
	31–35	4.5		2,000–5,000¥	34.7
	>35	0.8		50,001–10,000¥	46.9
				>10,000¥	6.1

## Data analysis results

### Reliability and validity analysis

Cronbach’s α coefficient was used to evaluate the reliability of the scale. Cronbach’s α coefficient of each latent variable was between 0.793 and 0.863, which was higher than 0.7; the combined reliability CR ranged from 0.797 to 0.863, both higher than the baseline of 0.7; the AVE ranged from 0.522 to 0.631, both higher than the baseline of 0.5. This indicates the reliability of the measurement scale in this study, as shown in Table 2.

**TABLE 2:** Reliability and validity analysis result.

Latent variable	Item	Factor load
	Before opening the blind box, I can’t judge the specific style of the product	0.768
Uncertainty α = 0.833	Before opening the blind box, I’m not sure whether the goods meet the expectations	0.868
CR = 0.836 AVE = 0.631	Before I open the blind box, I can’t be sure whether the goods are as expected	0.742
Functional value	The blind box has a beautiful appearance	0.751
α = 0.793	The blind box is exquisitely designed	0.740
CR = 0.836 AVE = 0.567	The quality of the blind box is very good	0.768
Emotional value	I enjoy the process of drawing the blind box	0.727
α = 0.864	Buying a blind box can bring me happiness	0.748
CR = 0.863 AVE = 0.560	I was looking forward to the process of extracting the blind box	0.755
Social value	Buying a blind box can strengthen my communication with my friends	0.718
α = 0.812	Buying blind boxes allows me to meet new friends	0.696
CR = 0.813	Buying a blind box shows my unique personality	0.737
AVE = 0.522	The blind box has become a bridge between me and other blind box players	0.737
Purchase intention	I will buy a blind box	0.726
α = 0.838	I will buy the blind box again	0.794
CR = 0.840	I look forward to buying the blind box again	0.788
AVE = 0.567	I am happy to recommend blind boxes to my friends	0.701

The discriminant validity test is conducted by comparing the correlation coefficients between the square root of the latent variable AVE and the latent variable, as shown in Table 3. According to Table 3, the AVE square root of latent variables is greater than the correlation coefficients between latent variables, indicating that the scale has good discriminant validity.

**TABLE 3:** Discriminant validity analysis result.

	Uncertainty	Functional value	Emotional value	Social value	Purchase intention
Uncertainty	0.794				
Functional value	–0.259	0.753			
Emotional value	0.443	–0.115	0.748		
Social value	0.339	–0.088	–0.150	0.722	
Purchase intention	0.198	0.216	0.403	0.323	0.753

### Structural model checking

In this study, Amos26.0 software was used to analyze the data of the research model. The results are shown in Table 4.

**TABLE 4:** Path test of the model.

	Standardized coefficients	SE	CR	Hypothesis
Uncertainty → Functional value	–0.259***	0.077	–4.096	Support
Uncertainty → Emotional value	0.443***	0.077	7.046	Support
Uncertainty → Social value	0.339***	0.076	5.317	Support
Functional value → Purchase intention	0.286***	0.057	4.749	Support
Emotional value → Purchase intention	0.392***	0.059	6.367	Support
Social value → Purchase intention	0.290***	0.059	4.799	Support

X^2^/df = 1.370, GFI = 0.948, AGFI = 0.932, NFI = 0.934, RMSEA = 0.031. ***indicates P < 0.001.

As shown in Table 4, uncertainty was negatively correlated with functional value (β = –0.259, *P* < 0.001), and positively correlated with emotional value (β = 0.443, *P* < 0.001) and social value (β = 0.339, *P* < 0.001). Hypotheses 1a, 1b, and 1c are supported. Functional value (β = 0.286, *P* < 0.001), emotional value (β = 0.392, *P* < 0.001), and social value (β = 0.290, *P* < 0.001) all had significant positive effects on customers’ purchase intention, among which, emotional value had the greatest influence on customers’ purchase intention. Hypotheses 2a, 2b, and 2c are all supported. In addition, the structural validity test indexes in the model all reached the standard, indicating that the data fit well with the theoretical model.

### Mediating effect test of the perceived value

Taking uncertainty as the independent variable, purchase intention as the dependent variable, and perceived value as the intermediary variable, using the process compiled by Hayes, 5,000 bootstrap times, 95% confidence interval, model 4 is selected for mediating effect test. The results show that (Table 5) the direct predictive effect of uncertainty on purchase intention is not significant (β = 0.0335, *t* = 0.6413, *P* > 0.05), indicating that the path coefficient between uncertainty and purchase intention is not significant in the direct effect model, and the perceived value fully mediates the impact of uncertainty on purchase intention.

**TABLE 5:** Mediating model test of perceived value.

	Functional value		Emotional value		Social value		Purchase intention	
				
Variable	β	*T*	β	*t*	β	*t*	β	*t*
Gender	0.036	0.723	–0.034	–0.709	0.998	0.658	–0.005	–0.109
Age	–0.027	–0.529	–0.091	–1.893	0.776	0.302	0.017	0.363
Uncertainty	–0.228	–4.471***	0.364	7.530***	0.002	5.374***	0.034	0.641
Functional value							0.224	4.768***
Emotional value							0.318	6.376***
Social value							0.234	4.922***
*R* ^2^	0.052		0.151		0.074		0.237	
*F*	6.838		22.021		9.839		19.066	

***Indicates P < 0.001.

With the addition of perceived value variables, uncertainty has a significant negative predictive effect on functional value (β = – 0.2282, *t* = 4.471, *P* < 0.01), while it has a significant positive predictive effect on emotional value(β = 0.002, *t* = 5.3735, *P* < 0.01) and social value (β = 0.3637, *t* = 7.5304, *P* < 0.01). Hypotheses 1a, 1b, and 1c are verified again. Functional value (β = 0.2243, *t* = 4.7678, *P* < 0.01), emotional value(β = 0.3178, *t* = 6.376, *P* < 0.01), and social value (β = 0.2336, *t* = 4.9222, *P* < 0.01) has a significant positive predictive effect on purchase intention, which verifies hypotheses 2a, 2b, and 2c again.

The percentile bootstrap method with deviation correction is used to further test the mediation effect. The 95% confidence interval of various effects is estimated by taking 5,000 bootstrap samples. If the confidence interval does not contain 0, the mediation effect is significant. The analysis results of intermediary effect show that the prediction effect of uncertainty on purchase intention is significant (β = 0.185, SE = 0.059, *P* < 0.01), and the 95% confidence interval is [0.0683,0.301]; the direct prediction effect is not significant (β = 0.0384, SE = 0.060, *P* > 0.05), 95% confidence interval [0.061,0.231]; the total mediating effect was significant (β = 0.146, SE = 0.043, *P* < 0.01), 95% confidence interval [–0.079,0.156]; the effect of each intermediary path is also significant. As shown in Table 6, the intermediary effect accounts for 79.2% of the total predicted effect. The above results show that functional value, emotional value, and social value play multiple mediating roles between uncertainty and purchase intention.

**TABLE 6:** Bootstrap analysis of mediation effect test.

Path	Effect	BootSE	BootLLCI	BootULCI
Total mediating effect	0.146	0.043	0.061	0.231
Uncertainty → Functional value → Purchase intention	–0.059	0.020	–0.102	–0.025
Uncertainty → Emotional value → Purchase intention	0.133	0.028	0.080	0.189
Uncertainty → Social value → Purchase intention	0.073	0.022	0.034	0.120

### The moderating effect of consumption purpose test: A multi-group analysis

To test the moderating effect of consumer intent, the overall sample was divided into pursuit process (*N* = 208) and pursuit outcome (*N* = 167), and Amos26.0 was used for multi-group analysis. There are three models: unconstrained model, measurement weight model, and structural weights model. By comparing the fit of different models, the unconstrained model was identified as a multi-group analysis model in this paper, and the fit indexes of this model were *X*^2/^*df* = 1.530, GFI = 0.878, AGFI = 0.862, NFI = 0.881, RMSEA = 0.038. The results show that different consumption purpose groups have a good fit for the conceptual model. The difference test of the first two models shows that there is no significant difference in the model as a whole (Δ*X*^2^ (14) = 19.061, *P* = 0.16 > 0.05), indicating that there is no significant difference in different grouping models, and multi-group analysis can be performed on the original model. The chi-square values of the unconstrained model and the measurement coefficient model are compared, and it is found that the chi-square values of the two models are significantly different (Δ*X*^2^ (20) = 44.013, *P* = 0.001 < 0.05), indicating that the path coefficients of structural equation models of different consumption target groups are significantly different. To further test the moderating effect, the CR index was used to compare the regression coefficients of consumers with different consumption objectives on the structural path. The results of the multi-group analysis are shown in Table 7.

**TABLE 7:** Moderating effect of consumption purpose.

	Process-oriented	Results-oriented	Coefficient difference	CR critical ratio	Regulatory effect
					
	Standardized coefficients	Standardized coefficients			
Uncertainty → Functional value	–0.185*	–0.431***	0.246	–2.098	Significant
Uncertainty → Emotional value	0.555***	0.333***	0.222	–0.35	Not significant
Uncertainty → Social value	0.332***	0.331**	0.001	–0.058	Not significant
Functional value → Purchase intention	0.237**	0.461***	–0.224	–2.763	Significant
Emotional value → Purchase intention	0.636***	0.351***	0.285	2.211	Significant
Social value → Purchase intention	0.247***	0.286**	–0.039	0.846	Not significant

**Indicates P < 0.01, *indicates P < 0.05, ***indicates P < 0.001.

As can be seen from Table 5, the negative effect of uncertainty on functional value and the positive effect of functional value on purchase intention is moderated by customer consumption. Hypotheses 3a and 4a are all supported. For process-oriented consumers, uncertainty has a stronger positive impact on emotional value, but this difference is not significant, hypothesis 3b does not support it. The reason may be that the customer who pays attention to the purpose can also experience the excitement and expectation brought by uncertainty in the process of box pumping.

The positive effect of emotional value on purchase intention is moderated by customer consumption. Hypothesis 4b is supported.

For consumers with different purposes, there is no significant difference in the coefficients of the two paths of the influence of uncertainty on social value and the influence of social value on purchase intention, and the moderating effect is not significant. Hypotheses 3c and 4c are not supported. The reason may be that in a blind box of consumption, regardless of whether to pursue a result, the player can share experiences, change kits, and harvest unexpected friendships.

## Conclusion and implications

### Conclusion

Uncertainty marketing is a way for enterprises to save marketing costs (Yan and Wu, 2021). Blind box, an emerging product favored by young people, has its core selling point of uncertainty. Understanding the positive role of uncertainty in blind box products can help enterprises better carry out marketing activities. This study examines the impact of uncertainty on blind box purchase intention from the perspective of perceived value, which is subdivided into functional value, emotional value, and social value. This study also discusses the moderating effect between uncertainty and perceived value, and between perceived value and customer purchase.

Our empirical data prove that perceived value plays a complete mediating role in the impact of uncertainty on purchase intention. Uncertainty has a significant negative impact on functional value and a significant positive impact on emotional and social values. Among them, uncertainty has the greatest impact on emotional value, which is consistent with the qualitative view of Zeng (2021) and Wang and Zhou (2021), but our study supplements empirical evidence. These results indicate that, in blind box consumption, uncertain gameplay mainly increases the emotional value of consumers, that is, curiosity, expectation, and satisfaction after the resolution of uncertainty in the process of box pumping, which affects the purchase intention of consumers. This also validates the views of Faraji-Rad and Pham (2017), who emphasize that the dependence of uncertainty on emotional input influences judgment and decision making. However, we further suggest that uncertainty not only increases dependence on emotional input but also increases emotional experience, which means that the effect of uncertainty is two-fold. The blind box of repeat purchase intention in the model, think social experience, unpredictable, and so on four factors together determine the blind box of repeat purchase intention (Yan and Wu, 2021). Uncertainty and social experience for the two variables are independent of each other, while in this paper, the conclusion shows that uncertainty has a positive influence on social value, which means the relationship between uncertainty and social experience is not independent.

When perceived value impacts purchase intention, the function value, emotional value, and social value significantly affect the blind box of purchase intention. Compared with functional value, emotional value and social value for a blind box of purchase intention were affected relatively stronger. This is inconsistent with the traditional conclusion of the influence of perceived value on purchase intention, which holds the view that perceived functional value plays the most important role in customer purchase (Sheth et al., 1991; Zhu et al., 2017).

In addition, this study examines the moderating effect of consumption purpose on uncertainty and perceived value, and perceived value and purchase intention. Customer consumption purpose significantly adjusts the impact of uncertainty on functional value, the functional value on purchase intention, and emotional value on purchase intention, while the moderating effects between uncertainty and emotional value, uncertainty and social value, social value, and purchase intention are not significant. In other words, results-oriented consumers pay more attention to functional value. Therefore, uncertainty has a greater negative impact on functional value. The view that different consumption objectives lead to different effects of uncertain marketing has been verified in both international and domestic markets (Shen et al., 2015; Shou et al., 2022), our study again applied and verified this conclusion in specific products. In addition, no matter what purpose consumers buy blind boxes, uncertainty can bring emotional value and social experience. However, because results-oriented customers have a clearer goal orientation, emotional and social values cannot become the decisive factor for them to buy.

### Implications

The conclusion of this paper has some enlightenment for academic research. First, this study is an important supplement to the application of uncertain marketing. Reducing consumer uncertainty is always the research focus, however, blind box as new products popular with young people; the uncertainty is the core factor to attract consumers to buy. Previous studies on the uncertainty of marketing focus on design promotion (Lee and Qiu, 2009; Laran and Tsiros, 2013; Shen et al., 2015), while the uncertainty in the blind box products is not involved in the promotion, but is a kind of daily sales. Taking blind box as the research object, this paper expands the positive effect theory of uncertainty in empirical research that verifies the explanatory power of perceived value on customer purchase. Second, this paper also expands the research on blind box products. Currently, most literature on the blind box is qualitative research (Wang and Zhou, 2021; Zeng, 2021). We provide new empirical evidence for blind box, an emerging hot product, to help us understand this new phenomenon. Finally, as the younger generation becomes the main consumer, we find new changes in the practical application of perceived value. Based on the importance of perceived value in marketing, this concept and its application have been repeatedly examined and deliberated by the academic community. According to Marshall’s (1920) theory of “rational economic man,” traditional research believes that functional value is the decisive factor influencing consumer purchase (Sheth et al., 1991), but the conclusion of this paper shows that for the younger generation of consumers, emotional value is gradually increasing in importance. Moreover, the drivers of perceived value are mostly analyzed from the perspective of profit and loss (Zeithaml, 1988). It is generally recognized that the driving factors of perceived value are mainly composed of deterministic factors such as product quality, service quality, and price (Parasuraman, 1997). Reducing profits and losses is also a way to improve perceived value, while the existence of uncertainty will undoubtedly make consumers unable to judge the real material of products, thus increasing profits and losses (Yang and Wang Yonggui, 2002). Contrary to the traditional view, our study concludes that uncertainty has a positive impact on perceived value, mainly due to the effect of uncertainty on emotion. Finally, Shen’s (2015) study verified the direct effect of consumption purpose on uncertain purchases by means of experimental manipulation. We also referred to his study to use consumption as a moderator variable, but our study found new findings. Consumption purpose does not directly regulate the influence of uncertainty on purchase intention, but mediates the influence of uncertainty on functional value, the functional value on purchase intention, and emotional value on purchase intention.

The findings of the study have implications for enterprise marketing. The results reveal the new preferences and new characteristics of the young generation of consumers and provide enterprises with an in-depth understanding of how uncertainty affects consumers’ purchase intention. First, according to the demographic characteristics of the blind box purchase samples, the buyers are mostly women, and the main age is between 18 and 25 years old. Enterprises can consider using the gender and age characteristics of consumers to segment the market when carrying out uncertain marketing. Second, research shows that in the blind box consumption, uncertainty mainly affects consumers emotional value, which is the key to deciding whether consumers purchase. Therefore, the enterprise when carrying on the uncertainty of marketing, should as far as possible to provide high quality, unique products, in order to enhance the consumers’ positive emotion and imagination. For example, in advertising and marketing, advertisers can strategically reserve part of the information through screen design, story design, and other ways to create an information gap to give consumers imagination space. At the same time, enterprises should also design stories for products and give them more emotional value. Third, according to the adjustment effect of consumption purpose, more attention should be focused on the consumption process during uncertain marketing, so that customers can have a more participatory experience and strengthen the fun of participation, and consumers are allowed to invest their time, emotion, and energy to create emotional memories between consumers and products. Meanwhile, hedonic products combined with a blind box marketing design may be better. Moreover, product design is also important, and young consumers may buy products simply because they are “pretty.”

### Limitations

Although our findings have a certain significance, our current research still has some limitations. First, we focus on the impact of uncertainty on purchase intention in blind box products, without considering other product types and scenarios; then, in the course of our research, we found the positive impact of blind box uncertain gameplay on social value. The mechanism between them and the follow-up results after players’ social sharing need to be studied.

## Data availability statement

The original contributions presented in this study are included in the article/supplementary material, further inquiries can be directed to the corresponding author.

## Ethics statement

Ethical review and approval was not required for the study on human participants in accordance with the local legislation and institutional requirements. Written informed consent from the participants’ legal guardian/next of kin was not required to participate in this study in accordance with the national legislation and the institutional requirements.

## Author contributions

Both authors listed have made a substantial, direct, and intellectual contribution to the work, and approved it for publication.
